# Looking for answers far away from the soma—the (un)known axonal functions of TDP-43, and their contribution to early NMJ disruption in ALS

**DOI:** 10.1186/s13024-023-00623-6

**Published:** 2023-05-31

**Authors:** Ariel Ionescu, Topaz Altman, Eran Perlson

**Affiliations:** 1 Department of Physiology and Pharmacology, Sackler Faculty of Medicine, Tel Aviv University, Room 605, Ramat Aviv, 69978 Tel Aviv, Israel; 2 Sagol School of Neuroscience, Tel-Aviv University, Tel-Aviv, Israel

**Keywords:** ALS, TDP-43, NMJ, Axon, Local synthesis, Condensates, Motor neuron

## Abstract

Axon degeneration and Neuromuscular Junction (NMJ) disruption are key pathologies in the fatal neurodegenerative disease Amyotrophic Lateral Sclerosis (ALS). Despite accumulating evidence that axons and NMJs are impacted at a very early stage of the disease, current knowledge about the mechanisms leading to their degeneration remains elusive. Cytoplasmic mislocalization and accumulation of the protein TDP-43 are considered key pathological hallmarks of ALS, as they occur in ~ 97% of ALS patients, both sporadic and familial. Recent studies have identified pathological accumulation of TDP-43 in intramuscular nerves of muscle biopsies collected from pre-diagnosed, early symptomatic ALS patients. These findings suggest a gain of function for TDP-43 in axons, which might facilitate early NMJ disruption. In this review, we dissect the process leading to axonal TDP-43 accumulation and phosphorylation, discuss the known and hypothesized roles TDP-43 plays in healthy axons, and review possible mechanisms that connect TDP-43 pathology to the axon and NMJ degeneration in ALS.

## Background

For many years, the molecular basis of neuromuscular junction (NMJ) disruption and motor neuron death in the fatal neurodegenerative disease Amyotrophic Lateral Sclerosis (ALS) has remained elusive. Recent fundamental discoveries in axonal biology in health and disease have advanced our understanding of the local and independent subcellular mechanisms that sustain motor neuron axonal function and integrity. Several of those discoveries also highlighted the pivotal contribution of axonal degeneration to the development and progression of ALS pathology. These may mark a new era for the development of novel therapeutic strategies, as well as diagnostic tools for this lethal disease.

TAR DNA Binding Protein (TDP-43) is a DNA and RNA binding protein that belongs to a group of heterogenous nuclear ribonuclear proteins (hnRNPs). TDP-43 was first discovered in 1995 by Ou and colleagues as a protein that binds the pyrimidine-rich motifs within the TAR DNA of human immunodeficiency virus-1 (HIV-1) [1]. It was demonstrated that TDP-43 represses the transcription of HIV-1 by altering or blocking the assembly of tat-responsive transcription complexes. A few years later, Buratti and colleagues were the first to describe an RNA-binding property for TDP-43, which mediates the exon 9 skipping of the CFTR mRNA during alternative splicing and has a fundamental role in the pathogenesis of several monosymptomatic and full forms of Cystic Fibrosis [2]. Ever since, numerous publications have reported various nuclear roles for TDP-43 in transcriptional and post-transcriptional regulation, including splicing of primary transcripts and miRNA biogenesis [3, 4]. The canonical TDP-43 protein includes several domains: Nuclear Localization Sequence (NLS), two RNA Recognition Motifs (RRM1 and RRM2) two Intrinsically Disordered Domains (IDRs), and a Low Complexity Domain (LCD; glycine-rich C’ terminal). Additionally, TDP-43 was shown to undergo multiple posttranslational modifications (PTMs) such as phosphorylation, acetylation, SUMOylation, and ubiquitination [5]. TDP-43 was also found to interact with other RNA-binding proteins, such as ubiquitin-2 (UBQLN2), FMRP, G3BP1, and TIA1 [6–12]. TDP-43 can also undergo proteolytic cleavage, leading to the formation of truncated, possibly pathological forms known as C-terminal fragments [13]. Mutations in the gene encoding for TDP-43, *TARDBP*, were reported in about 0.5% of ALS patients (familial and sporadic forms) [14, 15].

A primary interest in the non-nuclear roles of TDP-43 began shortly after discovering the presence of phosphorylated and ubiquitinated cytoplasmic inclusions of TDP-43 within motor neurons in the brains of patients with ALS and Frontotemporal Dementia (FTD) [16, 17]. Some of the inclusions seemed to include C-terminal fragments [16], which were also ubiquitinated and phosphorylated, although those were apparent only in brain tissue but not in spinal motor neurons [18]. In postmortem spinal cord samples from deceased ALS patients there was a clear indication of both nuclear clearance and cytoplasmic accumulation of TDP-43 in motor neurons [16]. This TDP-43 pathology was shown to be common for over ~ 97% of all ALS cases, regardless of their genetic background, and thus has almost completely rerouted ALS research towards gaining knowledge on the biology and pathobiology of TDP-43. This movement led to a long debate between researchers in the field who either advocate for the contribution of TDP-43 loss of nuclear function as a driver of ALS pathology, and those who favor the gain of toxic function driven by the aberrant mislocalization and accumulation of TDP-43 in the cytoplasm [19]. The high load of novel and comprehensive evidence on both ends indicates that the two pathologies co-exist, yet whether one is more detrimental than the other remains to be further discussed and studied. Deciphering the temporal relationship and the co-dependence between loss of TDP-43 from the nucleus and the appearance of cytoplasmic inclusions is key in understanding the disease development, and whether the formation of axonal TDP-43 inclusions is necessary and sufficient for motor neuron death and synaptic pathologies in ALS. Nevertheless, how those paradigms could explain the early pathological disruption of NMJs in ALS is still puzzling, although both loss and gain of TDP-43 function seem to play a role in axonal pathology [20, 21].

Motor neuron axons are the longest and most arborized ones in the nervous system. The volume of motor neuron axonal and synaptic cytoplasm (also known as axoplasm) exceeds that of the cell body by several orders of magnitude, and thus also its protein content. It has now been established that local protein synthesis and axonal transport are two essential mechanisms by which motor neurons use to regulate their axonal and synaptic proteome [22]. Alteration in both processes has been reported repeatedly in various animal models of ALS and in patient iPSC-derived motor neurons [20, 23–25]. Recently, we and others have reported that the accumulation and condensation of TDP-43 in ALS models and ALS patients are not limited to the cell body and can be detected early during the disease in peripheral motor neuron axons, concomitantly to the early NMJ disruption and axon degeneration [20, 26, 27]. Hence, the relation between these processes as well as the opportunity to intervene with the axonal TDP-43 accumulation should be extensively researched. In this review, we aim to discuss possible pathways by which TDP-43 can accumulate within motor neuron axons and synapses, dissect how the formation of TDP-43 axonal condensates disrupts typical axonal-specific roles of TDP-43, and how these can lead to ALS pathology. We will focus mainly on the disruption of RNA localization and local translation and the process by which their alteration may lead to the disease’s early degeneration of axons and NMJs.

## Part 1 – TDP-43 phosphorylation and its toxic role in axons of ALS patients

**Fig. 1:**
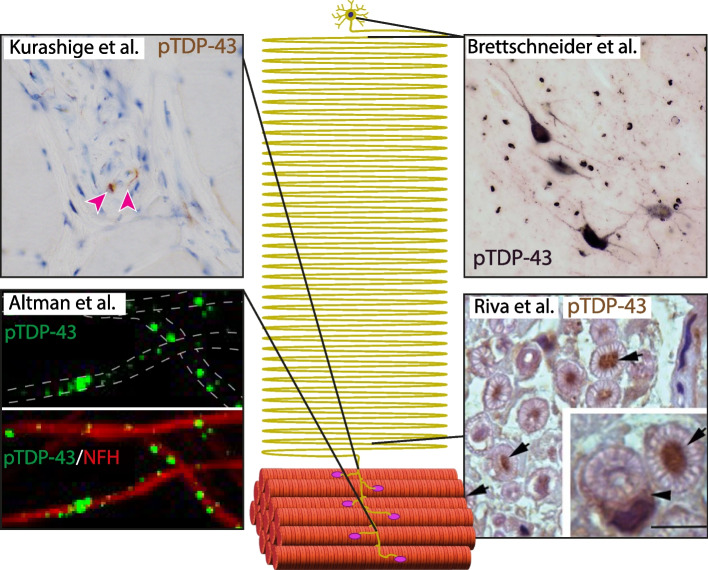
TDP-43 axonal pathology. An illustrative map of a motor neuron with annotations for previous observations of axonal TDP-43 pathologies in human patients. Brettschneider et al., reported TDP-43 pathology in spinal motor neurons and their neurites in postmortem spinal cord sections from ALS patients. Altman et al., Riva et al., and Kurashige et al., reported TDP-43 and phosphorylated-TDP-43 pathologies in distal nerve biopsies, and in intramuscular nerves within muscle biopsies of pre-diagnosed ALS patients. Arrowhead in image of Kurashige et al., indicates accumulation of pTDP-43 in intramuscular nerve bundles of sALS patient (Rabbit polyclonal pTDP-43 antibody; 22309–1-AP, pSer409/410, Proteintech). Kushirage et al., have also validated their findings with an additional pTDP-43 antibody (not shown; mouse monoclonal pTDP-43 antibody (TIP-PTD-M01, pSer409/410, CosmoBio). Arrows in image of Riva et al., indicate accumulation of pTDP-43 in large axons within motor nerve biopsies. Arrow in inset indicates accumulation of pTDP-43 in Schwann cells as well (Rabbit polyclonal pTDP-43 antibody; 22309–1-AP, pSer409/410, Proteintech). Authors have validated their observations with another antibody (not shown; anti-TDP-43 rabbit polyclonal (1:700, 10782–2-AP, Proteintech). Brettschnider et al., used two types of antibodies for detecting pTDP-43 in spinal cord samples: pTDP-43 pSer409/410 rabbit polyclonal antibody (CAC-TIP-PTD-P07; CosmoBio), and pTDP-43 pSer409/410 rat monoclonal antibody developed by Dr. M Neumann [30]. Altman et al., used two types of antibodies to detect TDP-43 and pTDP-43 accumulation in intramuscular nerves: Rabbit polyclonal pTDP-43 antibody; 22309–1-AP, pSer409/410, Proteintech, and Rabbit polyclonal TDP-43 antibody; 10782–2-AP, Proteintech. Images were adapted from original publications [20, 26, 27, 31]

ALS is a neurodegenerative disease that targets lower and upper motor neurons, with vast heterogeneity of proposed mechanisms that lead to toxicity in the disease. However, the existence of one common pathology has wide approval among experts, the presence of cytoplasmic inclusions of TDP-43 protein. Still, the notion that TDP-43 cytoplasmic inclusions can also accumulate in remote processes and facilitate local pathology in axons and synapses was until recently, a topic of controversy. This issue directly relates to the “dying back” theory, which depicts the NMJ and axons as the primary location for degeneration in the disease [28, 29]. Following this theory, which is supported mostly by animal models but also by some human ALS patient data [26–28], distal stress at the motor neuron axons and NMJ precedes the occurrence of cell death in the spinal cord and the brain. Recently, several research groups have successfully showed the presence of phosphorylated TDP-43 inclusions in peripheral motor axons [20, 26, 27] (Fig. 1). Here, we will present the collective evidence for axonal pTDP-43 presence in ALS, suggest possible mechanisms for how distal TDP-43 accumulation and phosphorylation occur and discuss if this pathology is solely an early marker of ALS or is a direct mediator of axonal toxicity.

### Evidence for the presence of axonal TDP-43 inclusions in ALS

A key fact, which is often being neglected when discussing TDP-43 pathology in ALS, is that most of the existing data regarding human ALS patient tissues are of hyperphosphorylated TDP-43 cytoplasmic inclusions. Early reports for the presence of TDP-43 phosphorylation (pTDP-43) came together with the finding that TDP-43 is a common pathology of ALS [16]. Further studies revealed the extent of this phenomena and characterized the shape, composition, and anatomical localization of pTDP-43 inclusions in different patients and disease stages [30–32]. While most focused on the identification of pTDP-43 in brain and spinal cord regions, very few tried to investigate peripheral nerve tissues. One pioneering work suggested the presence of dash-like pTDP-43 inclusions in proximal axons of cranial motor nerves VII and XII of post-mortem ALS patients, which was not apparent in controls [33]. Surprisingly, those protein inclusions stained negative for ubiquitin, leading to the hypothesis that their presence might be found at an early stage of the disease prior to extensive cellular damage. Several additional studies supported the finding of pTDP-43 condensates in motor nerve projections, although they mainly used post-mortem tissues and focused on proximal axons from cranial nerves and spinal cord [31, 32]. The first evidence for a more distal appearance of pTDP-43 condensates was a study of 19 Japanese sporadic ALS patients identifying pTDP-43 positive accumulation in pyramidal tracts, pre-synapses within the CNS, and most importantly, in distal axons of peripheral (in this case cranial) nerves [34]. Yet again, only post-mortem tissues were examined, missing the opportunity to test if pTDP-43 localization in neuronal projections is an early or late event in the course of the disease. In the last couple of years, several breakthrough studies decided to tackle this subject, and found that pTDP-43 condensates further distally in peripheral motor axons and at early symptomatic stages of ALS. The first study obtained samples from 102 patients suspected of ALS who underwent obturator motor-nerve biopsy and revealed that the appearance of pTDP-43 in axons is highly abundant in ALS patients, as 98.2% of patients had pTDP-43 inclusions in axons compared to 30.4% with non-ALS motor nerve defects [26]. Importantly, pTDP-43 inclusions were also found in samples from ALS patients with normal histopathological features, highlighting the early appearance of these in the ALS disease course. Two more studies have tried to observe this phenomenon closer to the nerve ending, by evaluating intra-muscular nerves from early symptomatic ALS patients who underwent muscle biopsy for diagnostic purposes. One study looked at a small population of 3 sALS patients and 5 controls and identified pTDP-43 condensates in distal axons which are co-localized with stress granule core protein G3BP1 [20]. The other was a large-scale cohort of 114 patients, which found pTDP-43 accumulation to be a highly sensitive marker for ALS identification. In this study, remarkably all muscle biopsies with localized intramuscular nerve bundles from ALS patients had evidence of pTDP-43 pathology, compared to none of the controls [27]. Additionally, it confirmed that pTDP-43 inclusion formation is an early pathological finding, which can be found even in early symptomatic ALS patients with mild functional motor neuron deficit at the time of biopsy. Interestingly, while pTDP-43 accumulation was exclusive to the ALS group in intramuscular nerves, in obturator nerve biopsies, it was detected in ~ 30% of patient biopsies with non-ALS conditions. This might be related to the site of biopsy, as the intra-muscular nerve represents the most distal axonal segment, possibly indicating that pathological pTDP-43 appearance is highly specific, especially at that region but less specific in more proximal axonal regions. Still, it is not well understood whether the early axonal appearance of pTDP-43 is solely a marker for axonal stress, or the driver for ALS axonal pathology. Also, little is known about the processes that regulate the localization of TDP-43 RNP granules to axons, as well as how those are phosphorylated.

### Possible causes for TDP-43 axonal accumulation

**Fig. 2:**
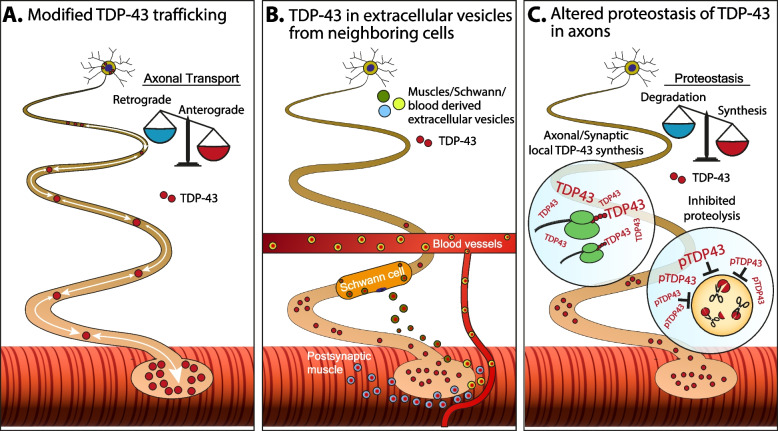
Proposed mechanisms of TDP-43 accumulation in axons. Illustration of motor units with the various possibilities for formation and accumulation of pathological TDP-43 in axons. **A** Alterations in axonal transport may result in a shift towards more anterograde or less retrograde transport of TDP-43 which will subsequently modify its local concentrations in axons and synapses. **B** TDP-43 spreading via extracellular vesicles. One way by which TDP-43 is disposed from cells is via extracellular vesicles. These TDP-43-containing vesicles could be removed from Schwann cells or skeletal muscles and be taken-up into neighboring axons, which cannot sustain protein overloads, and are less equipped with the appropriate machinery to cope with protein accumulation. **C** Alterations in proteostasis equilibrium driven by either non-regulated synthesis of TDP-43 in axons, or as been shown, by dysfunction in the process of TDP-43 proteolysis and clearance from axons due to its phosphorylated state

Several cellular mechanisms may explain the increase in local concentrations of proteins within axons: 1. Enhancement in cytoplasmic localization and axonal transport towards the nerve ending. 2. Decrease in axonal degradation and clearance. 3. Cell-to-cell protein transmission. 4. Local protein synthesis in axons. Although there is no concrete evidence as to which possibility is most likely in the case of TDP-43, several recent discoveries revealed few relevant pathways for this phenomenon in ALS (Fig. 2).

The first and most established mechanism is the mislocalization of TDP-43 to the cytoplasm and axons via defective nucleocytoplasmic transport. Several groups have now established proof that nucleocytoplasmic defects are involved in ALS pathology in both sporadic and familial ALS cases [35–37]. Due to those defects, the nuclear localization of TDP-43 is disrupted and it accumulates in the cytoplasm [38]. However, the sequence of events is not entirely clear. Can the formation of cytoplasmic or axonal TDP-43 condensates be the seed event that disrupts nucleocytoplasmic transport and facilitate the clearance of nuclear TDP-43? Or is it the other way around – nucleocytoplasmic defects are the initiating event? One hypothesis is that key proteins of the nuclear pore complex, Importin-α and Importin-β, bind the TDP-43 nuclear-localization-signal and prevent most of its cytoplasmic localization [39]. Interestingly, recent discoveries indicate that phosphorylation of TDP-43 in proximity to its nuclear-localization-signal prevents binding of Importins [39] and therefore can promote the cytoplasmic accumulation of the protein, thus suggesting that TDP-43 phosphorylation might be a key process that facilitates redistribution of the protein from the nucleus to the cytoplasm [40]. Additionally, a recent article by Khalil et al., indicates a new NLS-independent mechanism for TDP-43 cytoplasmic mislocalization, where the activity of karyopherin-β1 (KPNB1/Importin-β), increase the solubility of TDP-43 upon their recruitment to nucleoporin rich pTDP-43 condensates. Importantly, this activity was sufficient to prevent the formation of those condensates, acting as a molecular chaperone [41].

Additional mechanisms for TDP-43 accumulation in the cytoplasm are based on TDP-43 ability to go through anterograde axonal transport into axons as part of RNP granules for delivering mRNA to distal locations [42–44]. Therefore, if more TDP-43 is present in the cytoplasm, it is possible that increased anterograde or decreased retrograde axonal transport of TDP-43 to and from motor neurons axons and synapses will follow (Fig. 2A). Another pathway to be considered is the spreading of TDP-43 into axons by cell-to-cell protein propagation, which stems from several reports which discovered a distinct spreading pattern for TDP-43 aggregates [45, 46] (Fig. 2B). This possibility is based on the prion-like properties of TDP-43 [47, 48] and its ability to be secreted via exosomes [49] and through axon terminals [50]. One such possibility would be the transmission of TDP-43 or pTDP-43 from post-synaptic muscles into pre-synapses. Although a previous report has indicated that TDP-43 pathology is absent in ALS muscles [51], other reports have suggested muscular pTDP-43 pathology is apparent in a subset of ALS patients [52]. Additionally, the subsynaptic transcriptome and proteome at NMJs are unique compared to extrasynaptic regions [53] and thus, the possibility that TDP-43 is produced and transmitted at the postsynapse in muscles cannot be eliminated. Recently, pTDP-43 pathology was reported in Schwann cells as well as in axons within patient biopsies [26], yet whether these two coexist independently, or are the result of intercellular transmission from Schwann cells to axons (or vice versa) remains to be determined.

Lastly, and perhaps the most basic of all, the accumulation of TDP43 in axons could occur due to altered proteostasis in axons – either via inhibited proteolysis, or via local synthesis of TDP-43 (Fig. 2C). TDP-43, and specifically pathological truncated forms of it, known as C-terminal fragments, have been shown to create insoluble, phosphorylated and ubiquitinated cytoplasmic inclusions that can sequester cellular components critical for proteostasis [54]. It is currently unknown if truncated forms of TDP-43 are present in axons and can initiate similar events. Still, it is plausible that axonal accumulation of TDP-43 inclusions can result in altered protein degradation and clearance, even of TDP-43 itself. For example, this can lead to the prevention of proper protein folding which is normally carried out by specific heat shock proteins. The heat-shock chaperone HSPB1, was shown to prevent TDP-43 phase transition and therefore limit its ability to form cytoplasmic condensates. Upon their formation, those condensates are difficult to remove and are probable to be phosphorylated and generate toxicity [55]. Additional findings revealed that TDP-43 protein degradation is directly affected by chaperone mediated autophagy, mainly through Hsc-70 [56]. The axonal localization and function of those heat shock proteins is yet to be discovered, but it is possible that similar utilities apply there as well.

Alternatively, a recent report by Patel et al., has identified that the mRNAs of several RNA-binding proteins, such as Khsrp, are localized to peripheral nerve axons and are locally translated in response to injury [57]. TDP-43 is known to localize into axons and to carry mRNAs, including its own [39, 40, 53]. Additionally, detailed analysis of axonal transcriptome data from motor neurons revealed that TDP-43 mRNA is abundant in axons [58]. Therefore, the possibility that also TDP-43 can be locally synthesized in axons under certain conditions, such as in response to axonal or synaptic stress, should be further investigated.

Altogether, there are several conceivable pathways by which TDP-43 could aberrantly accumulate in axons. These pathways are distinct from one another but may explain the common axonal TDP-43 pathology among different ALS disease subtypes. Still, as the pathological consequence typically associates with TDP-43 phosphorylated form, it is highly possible that phosphorylation by itself plays a key role in TDP-43 localization and entrapment in axons.

### Factors regulating TDP-43 phosphorylation in axons

TDP-43 localization and function can be influenced by multiple factors, including protein expression, cleavage, and post translational modifications (PTMs) [5]. TDP-43 hosts several known influential PTMs, including phosphorylation [16, 30], ubiquitination [16], acetylation [16, 59, 60], SUMOylation [59, 60] and nitrosylation [61]. Although all of those have shown to be clinically relevant at a certain level [5], phosphorylation, and specifically hyperphosphorylation of the glycine-rich C-terminal domain, has been the most consistent marker of TDP-43 pathology in ALS patient brain and spinal cord [62]. As a demonstration of the high relevance of TDP-43 phosphorylation sites, at least 20 of TDP-43 related ALS mutations are localized at phosphorylation sites [62, 63]. Those ALS causing mutations have been found to alter protein localization, interactions with other proteins, clearance capacity and a tendency for phase separation [63–66]. As for the role of TDP-43 phosphorylation in mediating toxicity, a common hypothesis is that enhancing phosphorylation of TDP-43 is a key event leading to neurodegeneration in TDP-43 proteinopathies and specifically ALS [62]. Still, alternative explanations exist as well. It was previously suggested that TDP-43 phosphorylation also possesses positive neuroprotective impact due to interference with liquid–liquid phase separation [5, 67], which precedes protein aggregation and acts as a compensatory mechanism to increase protein solubility. Indeed, phosphorylation, as well as other PTMs on TDP-43, most probably possess important physiological functions, regardless of the pathological modifications [5]. This discrepancy may be explained by considering that pathological phosphorylation and accumulation of TDP-43 may arise from a dysregulation in the location, timing, or intensity of the physiological phosphorylation, or dephosphorylation of TDP-43. This interplay between the physiological and pathological roles of TDP-43 phosphorylation requires further study, possibly via the generation of phospho-dead/phospho-mimics mutants, inhibition of endogenous kinases/phosphatase and nevertheless improving the specificity in detection of pTDP-43 forms. Furthermore, the role of TDP-43 phosphorylation in axons should be studied as well to explore whether the factors which regulate it are axonal specific or similar to the ones of the cell body.

Like other phosphorylated proteins, there is an enzymatic pathway coordinating TDP-43 phosphorylation, which is mediated by kinases and phosphatases. Five known kinases can directly phosphorylate TDP-43: Casein kinases 1 and 2 (CK1, CK2), Tau tubulin kinases 1 and 2 (TTBK1, TTBK2) and Cell division cycle 7 (CDC7) kinase [62]. Among those, it was demonstrated that CK1 activation could directly induce TDP-43 cytoplasmic mislocalization, aggregate formation and neurotoxicity [68]. Interestingly, it was found that CK1 expression is tightly linked to TDP-43 phosphorylation in sporadic ALS patients' spinal cords, suggesting clinical relevance for this protein [69]. Furthermore, blocking CK1 activity by pharmacological compounds has shown a neuroprotective effect and led to the recovery of TDP-43 localization and function in vivo and in vitro [70]. Recently, it was shown that inhibition of CK1 but not TTBK1/2 could be modified by CHMP2B, a protein associated with ALS and FTD, thus causing decreased TDP-43 phosphorylation and neuroprotection [71]. Other TDP-43 kinases, TTBK1/2 [72, 73] and CDC7 [74] were found in several studies to cause TDP-43 associated neurodegeneration upon their activation, making them a promising target for the prevention of TDP-43 phosphorylation as well.

With regard to the opposite regulation of TDP-43 phosphorylation, the phosphatases Calcineurin, PP1 and PP2 have been shown to have a direct impact on TDP-43 phosphorylation [62]. It was shown that calcineurin is inversely regulated by TDP-43 expression [75], interacts with TDP-43 in samples from patient brains, and its activity is decreased in brain lysates from ALS patients [76]. Additionally, in *C.elegans* models of TDP-43 proteinopathy, genetic deletion of calcineurin homolog gene tax-6 can lead to excess accumulation of pTDP-43 and motor dysfunction, while treatment with the calcineurin inhibitor tacrolimus rescued the motor phenotype [77]. As for PP1 and PP2, it was demonstrated that they immunoprecipitate with TDP-43 and that PP1 but not PP2 expression can suppress TDP-43 phosphorylation [78].

Still, regarding the activity of those kinases and phosphatases in the regulation of TDP-43 phosphorylation in axons and its functional outcomes, data is sparse at best. One possibility is that axonal kinase activity might be influenced indirectly from TDP-43 phosphorylation by itself. For example, CK2, a kinase known to phosphorylate TDP-43, was found to enhance axon growth by phosphorylating G3BP1 [79]. G3BP1 phosphorylation, in contrast to TDP-43 phosphorylation, is known to lead to the disassembly of axonal RNP granules and subsequently to increase intra axonal protein synthesis [80]. As pTDP-43 was shown to enhance G3BP1-mediated axonal translation inhibition [20], it is possible that indirect influence of pTDP-43 activity can inhibit CK2 activity, leading to increased axonal condensate formation and toxicity. Thus, various mechanisms influencing TDP-43 phosphorylation exist, and future research will aim to investigate where TDP-43 is phosphorylated and how the regulation of TDP-43 phosphorylation can impact axon degeneration.

## Part 2—Possible mechanisms by which TDP-43 implicates axonal toxicity

Two pivotal questions which remain open are how TDP-43 is capable of mediating excessive damage to motor neuron axons and NMJs, and if there is higher vulnerability of those distal segments to TDP-43 condensation compared to cell bodies. To address those issues, it is first necessary to understand the unique properties of motor neurons and the roles TDP-43 has in supporting their growth and maintenance. Motor neuron axons are of the longest and most arborized axons in the human body. To coordinate complex spatiotemporal processes within their highly compartmentalized cytoplasm, neurons have adapted key mechanisms such as axonal transport of essential proteins [81, 82], organelles [83, 84], mRNAs [85], and an ability to locally translate mRNAs into proteins [22]. These two processes are co-dependent on each other, as axonal transport shuttles mRNA into axons, while the local synthesis of several motor protein subunits and adaptors [86, 87], as well as proteins maintaining the microtubule infrastructure [88, 89] are required for rapid and efficient response to both intra and extracellular changes. mRNA shuttling into axons, their local translation, and their stabilization, are tightly regulated by RNA-binding proteins (RBP), which recognize and bind to specific nucleic-acid motifs [85] within RNAs to assemble RNP granules [90, 91]. RNP granules have been shown to halt translation of the mRNA within them during their shuttling and localization, supporting mRNA polarization and local translation [91]. TDP-43 contains two RRMs (RRM1, RRM2) and thus can function as an RBP, mainly through its high affinity to GU rich sequences within RRM1 [92]. Additionally, TDP-43 contains LCD at its C-terminus which drives the formation of these RNP granules [66]. Despite the fact that it is primarily nuclear, when localized in the cell cytoplasm, TDP-43 is actively transported into axons [42, 43]. TDP-43 interactome analysis revealed its binding to additional proteins associated with RNA trafficking and axon localization, including several RBPs such as Staufen-1, FMRP, G3BP1 and SMN [8, 9, 93–95].

Owing to the tremendous efforts invested in identifying TDP-43 interactors and TDP-43-bound mRNA, our knowledge of the cytoplasmic role of TDP-43 and more specifically in axons advanced in recent years. As TDP-43 is strongly associated with the formation of cytoplasmic and axonal inclusions in ALS, data from studies on these pathological forms assisted to reveal the normal context of TDP-43 at those subcellular environments. Various approaches were used to characterize the TDP-43-bound, and TDP-43-associated RNA populations in the nucleus, as well as in the cytoplasm [96, 97]. Sephton and colleagues performed TDP-43 RNA-Immunoprecipitation (RIP) from primary rat cortical neurons, followed by deep sequencing, and identified that TDP-43 binds several protein-coding RNAs involved in neurodegeneration including its own mRNA, FUS/TLS mRNA, progranulin, Ataxin1/2, and TAU. Using gene-ontology, the authors also revealed that TDP-43 binds groups of mRNAs related to synaptic function, mRNA metabolism, and neurodevelopment. Interestingly, they report that the transcripts which co-precipitate with TDP-43, contain binding motifs for the RBP PTBP2, suggesting TDP-43 and PTBP2 may collaborate in regulating TDP-43 mRNA targets.

Due to the spatial complexity of motor neurons, and considering the axonal pathology in ALS, a key unresolved question is identifying the RNAs associated with TDP-43 also in axons and NMJs. Alami and colleagues have identified that TDP-43 is a component of axonal mRNA granules where it binds and shuttles RNA [42]. TDP-43 was shown to interact with its own mRNA in an autoregulatory fashion [98], as well as with mRNAs involved in axonal cytoskeleton (*Nefl, Map1b*) and mitostasis process (*Cox4i1, ATP5A1*) [20]. Moreover, a recent report also demonstrated that the knock-down of TDP-43 disrupts axonal transcriptome and impairs local translation [21]. Recently, Liao and colleagues have suggested that RNP granules interact with Annexin A11 to hitchhike lysosomes and traffic long distances in axons [44], while others have indicated that nuclear-encoded mitochondrial mRNA transports into axons on top of mitochondria [99, 100]. Cioni et al., have reported that RNP granules and ribosomes associate with late endosomes that anchor axonal mitochondria and serve as a platform for local synthesis of essential mitochondrial proteins [101]. TDP-43 also associates with mitochondria [102] and was shown on several occasions to bind nuclear encoded mitochondrial mRNAs [20, 102]. Hence, through direct and indirect interactions, TDP-43 serves a critical function in regulating the localization and translation of mitochondria related mRNAs in axons, thus influencing distal mitochondria maintenance. These discoveries may serve to explain the many studies demonstrating dysfunctions in mitochondria activity and transport which are considered to be key pathologies in ALS and were also described in TDP-43 mutant models [20, 102–104].

### Regulation of TDP-43 on local protein synthesis

Many publications in the recent decade have established that axons and synapses rely on local protein synthesis in order to develop, function, respond to changes, and maintain themselves [20, 86, 87, 101, 105, 106]. Yet, our understanding of the mechanisms that promote and regulate RNA localization and local translation at a specific synapse or in specific segments within axons remains unsatisfactory.

Accumulating evidence indicates that these processes are controlled by the dynamic assembly and dissolution of RNP granules [107, 108]. The formation of these granules, and their disassembly were shown to regulate RNA translation in axons [20, 80, 109]. The identification of TDP-43, and also other important RBPs such as FUS [110], in axons and in the pre-synapse at NMJs imply on their potential role in regulating local synthesis [23, 43, 96]. Gopal and colleagues indicated that TDP-43 could form phase-separated, droplet-like RNP granules in axons [111]. These granules contain RNA, and appear to be dynamic in composition, interact with each other, and maintain an equilibrium with the cytoplasmic pool of TDP-43. Interestingly, these traits were more prominent in mid axons compared to proximal axons, suggesting a spatial role for TDP-43 granules in motor neurons. Upon cellular stress in the neuronal cell body, as well as in other eukaryotic cells, TDP-43 is recruited to G3BP and TIA1 RNPs which promote the assembly of stress-granules [6]. Stress granules are macromolecular membranelles organelles which sequester mRNAs, as well as additional RBPs and translation factors and facilitate a global reduction in protein synthesis to allow the cells to prevail and recover [93, 112, 113]. Russo and colleagues have shown that TDP-43 RNA granules also include translation machinery, an interaction that is mediated by the ribosomal scaffold protein RACK1 [114]. The authors also demonstrate that RACK1 promotes the recruitment of TDP-43-polysome complexes onto stress granules, and that increase in the cytoplasmic levels of TDP-43 leads to a greater association of TDP-43 with ribosomal machinery, and to a subsequent reduction in protein synthesis. By contrast, Neelagandan and colleagues indicated that overexpression of human wild-type TDP-43 or mutant TDP-43^A315T^ in motor neuron-like cortical neurons does not affect global protein synthesis, however, it does enhance the translation of specific transcripts, including Camta1 and Mig12, and that overexpression of mutant TDP-43^A315T^ also enhances translation of Dennda4 mRNA [115]. In *Drosophila Melanogaster* models, Coyne et al., have shown that TDP-43 and drosophila FMRP colocalize together in PABP-positive stress granules in cultured neurons from TDP-43 overexpression model flies. The authors further show that overexpression of TDP-43 inhibits the translation of drosophila NMJ-associated *futsch* mRNA by its sequestration in these stress granules [116]. Hence, TDP-43 seems to act as a transcript-specific translational switch, yet the context and conditions controlling it are unknown. Recently, Briese et al., have described that loss of axonal TDP-43 alters axonal transcriptome and disrupts axonal protein synthesis [21]. Similar traits were detected in other forms of ALS where FUS, another RNP, is mutated [23], hinting for a common spatial specific role for TDP-43 and ALS-related RNPs in axons to support local protein synthesis and maintain axonal health.

Axonal and synaptic mitochondria have been shown to depend on local protein synthesis of nuclear-encoded mitochondrial mRNAs in order to properly function [20, 100, 117–119]. In our recent publication, we found that accumulation of TDP-43 leads to an overall decrease in mitochondrial proteins in sciatic axoplasm of TDP-43ΔNLS mice [20]. TDP-43-RNA immunoprecipitation (RIP) validated that indeed TDP-43 binds nuclear-encoded mitochondrial gene transcripts, specifically ATP5A1, COX4i and Ndufa4. Loss of TDP-43 leads to a complementary outcome, and resulted in dysregulation of transcripts associated with mitochondrial, as well as synaptic function [21]. Indeed, both accumulation of TDP-43, loss of TDP-43, and mutations in TDP-43 lead to mitochondrial dysfunction in axons, underlining the fundamental role of axonal TDP-43 in health and disease [20, 21, 103, 120].

Following mitochondrial ones, transcripts encoding ribosomal subunits were also reported to occupy large proportions of axonal transcriptome [121, 122]. Recently, a broader contribution of TDP-43 to the axonal protein synthesis was described by Nagano et al. This group reported that TDP-43 binds and shuttles mRNAs encoding ribosomal proteins [123]. The localization and local translation of ribosomal protein mRNAs is essential for renewing the axonal ribosomal pool and is required for proper local protein synthesis. Interestingly, accumulation of TDP-43 in TDP-43ΔNLS mouse sciatic nerves, coincided with an increase in ribosomal subunit proteins [20].

Another layer of regulation over translation is achieved by microRNAs (miRNA). miRNAs are short, 20–22 nucleotide non-coding RNA that mediate posttranscriptional silencing of their tens to hundreds of target mRNAs, some of which encode protein families that promote a specific function or pathway [124]. As such, miRNAs participate in the regulation of RNA translation at subcellular resolution, including in axons, where we and others have detected the RNAi machinery [58, 125–128]. Similarly to mRNAs, precursor miRNA were also recently described to hitchhike late endosomes/lysosomes in distal axons, and mature in response to extrinsic cues such as Sema3A [129, 130]. Kawahara et al., and others have indicated that TDP-43 promotes the biogenesis of miRNA at several phases. While nuclear TDP-43 binds both Drosha as well as several primary miRNAs, cytoplasmic TDP-43 binds pre-miRNA at their terminal loop and interacts with the Dicer complex [131, 132]. These functions were shown to be vital for proper neuronal growth. More recently, Perez-Colasante et al., identified the miRNAs that TDP-43 binds and ones that may be affected by its ALS-associated cytoplasmic mislocalization and accumulation [133]. Lastly, miRNAs and the RNA-induced Silencing Complex member Argonaut-2 (Ago2) [134, 135] were described to be recruited to stress granules and interact with stress granule proteins upon stress, suggesting a link between their interaction with TDP-43, and its axonal function in regulating translation.

Collectively, this evidence marks cytoplasmic and axonal TDP-43 as a regulator of the axonal transcriptome and proteome. Yet, the mechanisms that regulate TDP-43 and control its ability to perform distinct properties over space and time in motor neuron axons and synapses remain to be determined. The post-transcriptional modifications which TDP-43 has been described to undergo, like phosphorylation, are one possible explanation for how TDP-43 switches between its different functions and localizations (nucleus/cytoplasm/axons), but may also cause neurodegeneration when are aberrantly made in space, time and intensity. It is highly important to understand how those switches occur, and even more so how we can interfere with them early enough to slow or prevent motor neuron degeneration in ALS.

## Part 3 – Clinical implications of TDP-43 mislocalization in axons on ALS pathology

As years go by, scientific discoveries have promoted our understanding of ALS disease etiology, genetics, pathology and progression. Still, those achievements did not yet translate into the clinic. Therefore, it is necessary to ask, are we “looking under the lamp”?. Meaning, are current therapeutic interventions developed for ALS the most likely to succeed or the most available for testing. One key point regards the relative overlook of axonal projections and NMJs when trying to analyze ALS pathology. Until recently, the knowledge about ALS pathology was based mostly on histological sections of post-mortem CNS tissues, such as the brain and spinal cord [16, 30, 33]. Isolation of tissues from early symptomatic patients was considered complicated ethically and practically, and as a result, most patient data was not obtained from peripheral nerves and at early stages. Thus, the crucial majority of studies regarding TDP-43 based pathology and mechanistic implications were gained from cell bodies without assessing axonal projections. Furthermore, even when observing primary motor neurons or IPSC-derived motor neurons, some popular hypotheses were validated only on cell bodies without confirming their influence on axons and NMJs. However, the new discoveries of TDP-43 pathology in distal axonal projections which appear already in early stages of the disease [26, 27], suggest that this approach did not provide a complete picture. This novel data directs ALS researchers to acknowledge also the motor neurons' periphery, which remained understudied mostly due to technical reasons. Thus, the implications of TDP-43 axonal localization on motor neuron toxicity in ALS require much more attention. Many mechanisms were previously suggested to explain the effect of TDP-43 aggregation, spanning from alterations in mitochondria activity [20, 104, 136], axonal transport [137], protein translation [20, 123], stress granule formation [111, 138], aberrant splicing and genetic instability [139, 140], nucleocytoplasmic defects [37] and more. However, the knowledge about the relevance of those toxic effects on axonal and NMJ viability is less studied. Thus, a deeper look is required at how existing known pathways of TDP-43 toxicity converge to form the disease [141], especially in distal neuronal compartments.

### Implications of axonal pTDP-43 condensate formation

An important topic, which was discussed earlier, is the ability of TDP-43 protein to form cytoplasmic condensates. Still, little is known regarding the presence of those condensates in axons and their ability to form and dissolve in axons of ALS models. Over the last 10 years, a liquid-to-solid phase transition has become a central mechanism in ALS pathology [142–144]. Multiple studies have suggested mechanisms by which the propensity of TDP-43 and other ALS-relevant RNPs such as FUS to form devastating insoluble aggregates is critical for driving toxicity [143, 145]. This process was shown to cause aberrant mRNA splicing [140], inhibit protein synthesis [20, 80], sequester RNA and impair its metabolism [143]. Those processes are deeply related to TDP-43 protein structure which contains an LCD, making it prone to form condensed protein-RNA structures [144, 146]. Upon cytoplasmic localization, TDP-43 was shown to colocalize with known RNP granule core proteins such as G3BP1 and TIA1 [6, 147]. However, current knowledge about the relevance of those structures in ALS and in axons is concerningly missing. Only a handful of studies have tried to unravel the formation of those stress granules in vivo at all, and even scarcer data was obtained using ALS in vivo models and from ALS patients. One pilot study showed that ALS model mice have indeed a higher tendency to form stress granules in neurons and a decreased ability to degrade such structures in vivo [148]. However, other studies with TDP-43 models saw the opposite effect [149]. Therefore, the lack of evidence prevents us from solidifying the assumed roles of axonal condensates in ALS pathology, even though it is highly plausible that better technical abilities to preserve RNP condensate formation in vivo will aid in solving this intriguing topic.

Additionally, the function of RNP condensates in axons and synapses is also understudied. Gopal et al. revealed that axonal TDP-43 has influence on the solubility of RNP granules, and mutations in TDP-43 can influence its axonal transport [111]. A recent study revealed that upon stress or injury, axons are able to release RNA from such RNP condensates to allow local protein translation [80]. Some key stress granule proteins, such as G3BP1, have been found to directly influence this process, by acting as an inhibitor for intra-axonal protein synthesis. Importantly, the breakdown of those G3BP1 granules in axons was shown to be critical for axon regeneration and the prevention of it led to axon degeneration following injury [80]. Interestingly, interfering with G3BP1 axonal RNP condensate formation using G3BP1 mimicking peptides was shown to enhance axon regeneration [80], prevent axon degeneration and preserve NMJ function [20]. Therefore, it is possible that finding molecules that will interfere with specific types of pathological RNP condensate formation in axons can serve as a novel therapeutic avenue in ALS.

### Future therapeutic approaches in ALS

Targeting motor neuron axons and NMJs with novel therapies early in the disease course might prove to be beneficial for some additional reasons. One possible advantage can be the increased pharmacological accessibility of peripheral nerves. Traditionally, it is difficult to develop and administer therapies for neurological diseases, due to the complex process of efficiently crossing the blood–brain barrier in order to reach adequate drug concentrations in the cerebrospinal fluid. If indeed the formation of pTDP-43 disease-causing condensates is initiated in peripheral axons and NMJs, it is possible that blocking this process can delay the disease or even prevent it from affecting the spinal cord where motor neuron cell bodies are located. There are already known compounds that were shown to inhibit TDP-43 phosphorylation and even to block condensate formation which might be used [150]. However, it is important to take this hypothesis with a grain of salt, as little is known about the possible side effects of such treatments on other cell types in the body. Additionally, this approach will be insufficient, mainly due its limited access to the CNS, which is also affected in the disease. We are still very far from initiating trials with TDP-43 condensate modifying therapies. Still, the lack of efficient treatment for ALS at the moment calls for developing an alternative approach. The option of targeting peripheral axons is not the only important aspect, but it may ease the way for the initiation of new clinical trials, hoping to bring novel treatments that will specifically affect motor neurons and alter ALS disease course.

## Conclusions

**Fig. 3:**
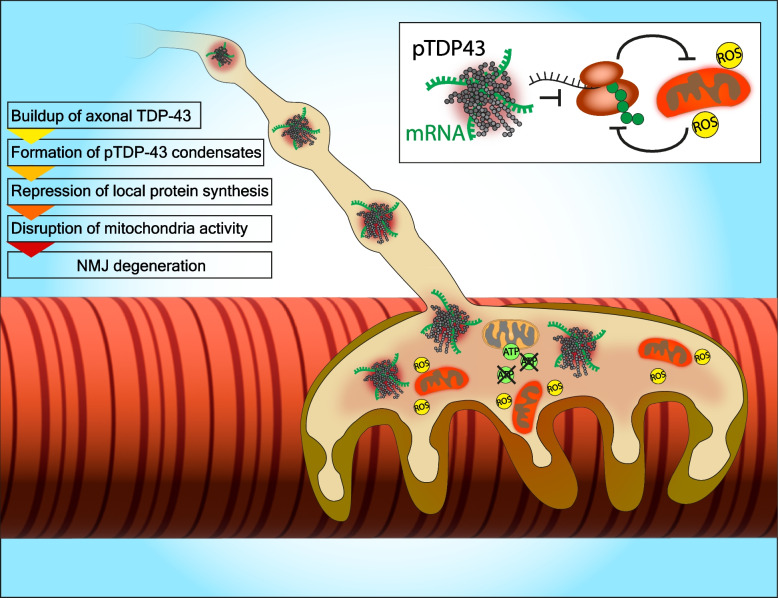
Proposed pathological mechanism of TDP-43 in axons/NMJs. Recent evidence on both the biological and pathological roles of axonal TDP-43 imply that TDP-43 accumulates in remote axons and presynaptic compartments of motor neurons and forms pTDP-43 condensates. Those structures sequester mRNAs critical for local synthesis of mitochondrial and ribosomal proteins. The disruption in local protein synthesis of these initiates a vicious cycle between mitochondria dysfunction, generation of Reactive Oxygen Species (ROS), and further impairment of local protein synthesis. This specifically impacts the NMJ due to the strict dependency of the NMJ on those processes, thus facilitating NMJ and axon degeneration

The novel finding regarding the presence of pTDP-43 pathology in peripheral motor neuron axons of ALS patients provides an exciting new perspective for understanding ALS pathology and etiology, as well as an avenue for developing new therapeutic approaches. Together with recent discoveries regarding the importance of TDP-43 in axonal health and the implications of its phosphorylation on RNA biology, local protein synthesis, and mitochondrial viability, an urgent need for timely research is at hand. One possible hypothesis which will need further proof is that the initial step in the disease is enhanced axonal localization of TDP-43 at distal motor nerves. This process can lead to a buildup of pTDP-43 condensates in the NMJ, which in turn facilitates repression in local protein synthesis, mainly of mitochondrial and ribosomal proteins. The following result is the failure of the NMJ to maintain enough functioning mitochondria and ATP, resulting in the accumulation of reactive oxygen species and NMJ degeneration, delivering a retrograde signal causing motor neuron cell death (Fig. 3). It is early to determine the exact order of events, but to do that, future research must determine if the effects studied for TDP-43 in the motor neuron cell body and peri-nuclear cytoplasm occur in distal neuronal compartments and when. The next stage will be to try and utilize this new knowledge both as an anchor for therapeutic development and as a call for ALS researchers to enhance their studies of axonal and synaptic biology. Hopefully, those joined forces will be able to look for the convergence of ALS mechanisms and their effect on the peripheral nervous system. This will lead to mechanism-based therapy that will aid ALS patients in their struggles.

## Data Availability

Not applicable.

## Abbreviations

ALS: Amyotrophic Lateral Sclerosis
FTD: Frontotemporal Dementia
NMJ: Neuromuscular Junction
pTDP-43: Phosphorylated TDP-43
RNP: Ribonucleoprotein
RBP: RNA binding protein
RIP: RNA Immunoprecipitation
BBB: Blood Brain Barrier
CNS: Central Nervous System
LCD: Low Complexity Domain
NLS: Nuclear Localization Sequence
NES: Nuclear Export Sequence
RRM: RNA Recognition Motif
PTM: Posttranscriptional Modifications

## References

[1] Ou SH, Wu F, Harrich D, García-Martínez LF, Gaynor RB (1995). Cloning and characterization of a novel cellular protein, TDP-43, that binds to human immunodeficiency virus type 1 TAR DNA sequence motifs. J Virol.

[2] Buratti E (2001). Nuclear factor TDP-43 and SR proteins promote in vitro and in vivo CFTR exon 9 skipping. EMBO J.

[3] Ederle H, Dormann D (2017). TDP-43 and FUS en route from the nucleus to the cytoplasm. FEBS Lett.

[4] Buratti E, Baralle FE (2008). Multiple roles of TDP-43 in gene expression, splicing regulation, and human disease. Front Biosci.

[5] Sternburg EL, Gruijs da Silva LA, Dormann D (2022). Post-translational modifications on RNA-binding proteins: accelerators, brakes, or passengers in neurodegeneration?. Trends Biochem Sci.

[6] McDonald KK (2011). TAR DNA-binding protein 43 (TDP-43) regulates stress granule dynamics via differential regulation of G3BP and TIA-1. Hum Mol Genet.

[7] Kanai Y, Dohmae N, Hirokawa N (2004). Kinesin transports RNA: Isolation and characterization of an RNA-transporting granule. Neuron.

[8] Freibaum BD, Chitta RK, High AA, Taylor JP (2010). Global analysis of TDP-43 interacting proteins reveals strong association with RNA splicing and translation machinery. J Proteome Res.

[9] Ling SC, Polymenidou M, Cleveland DW (2013). Converging mechanisms in als and FTD: Disrupted RNA and protein homeostasis. Neuron.

[10] Cassel JA, Reitz AB (2013). Ubiquilin-2 (UBQLN2) binds with high affinity to the C-terminal region of TDP-43 and modulates TDP-43 levels in H4 cells: Characterization of inhibition by nucleic acids and 4-aminoquinolines. Biochim Biophys Acta Proteins Proteom.

[11] François-Moutal L (2019). Structural Insights Into TDP-43 and Effects of Post-translational Modifications. Front Mol Neurosci.

[12] Giambruno R, Grzybowska EA, Fawzi NL, Dormann D (2022). Editorial: The Role of Protein Post-Translational Modifications in Protein-RNA Interactions and RNP Assemblies. Front Mol Biosci.

[13] Berning BA, Walker AK (2019). The pathobiology of TDP-43 C-terminal fragments in ALS and FTLD. Front Neurosci.

[14] Sreedharan J (2008). TDP-43 mutations in familial and sporadic amyotrophic lateral sclerosis. Science.

[15] Su XW, Broach JR, Connor JR, Gerhard GS, Simmons Z (2014). Genetic heterogeneity of amyotrophic lateral sclerosis: Implications for clinical practice and research. Muscle Nerve.

[16] Neumann M (2006). Ubiquitinated TDP-43 in frontotemporal lobar degeneration and amyotrophic lateral sclerosis. Science.

[17] Arai T (2006). TDP-43 is a component of ubiquitin-positive tau-negative inclusions in frontotemporal lobar degeneration and amyotrophic lateral sclerosis. Biochem Biophys Res Commun.

[18] Igaz LM (2008). Enrichment of C-terminal fragments in TAR DNA-binding protein-43 cytoplasmic inclusions in brain but not in spinal cord of frontotemporal lobar degeneration and amyotrophic lateral sclerosis. Am J Pathol.

[19] Suk TR, Rousseaux MWC (2020). The role of TDP-43 mislocalization in amyotrophic lateral sclerosis. Mol Neurodegener.

[20] Altman T, et al. Axonal TDP-43 condensates drive neuromuscular junction disruption through inhibition of local synthesis of nuclear encoded mitochondrial proteins. 2021;12:1–17. 10.1038/s41467-021-27221-8 PMC8617040 34824257

[21] Briese M (2020). Loss of Tdp-43 disrupts the axonal transcriptome of motoneurons accompanied by impaired axonal translation and mitochondria function. Acta Neuropathol Commun.

[22] Holt CE, Martin KC, Schuman EM (2019). Local translation in neurons: visualization and function. Nat Struct Mol Biol.

[23] López-Erauskin J (2018). ALS/FTD-Linked Mutation in FUS Suppresses Intra-axonal Protein Synthesis and Drives Disease Without Nuclear Loss-of-Function of FUS. Neuron.

[24] Ionescu A (2019). Targeting the Sigma-1 Receptor via Pridopidine Ameliorates Central Features of ALS Pathology in a SOD1 G93A Model. Cell Death Dis.

[25] Bilsland LG (2010). Deficits in axonal transport precede ALS symptoms in vivo. Proc Natl Acad Sci U S A.

[26] Riva N (2022). Phosphorylated TDP-43 aggregates in peripheral motor nerves of patients with amyotrophic lateral sclerosis. Brain.

[27] Kurashige T (2022). TDP-43 Accumulation Within Intramuscular Nerve Bundles of Patients With Amyotrophic Lateral Sclerosis. JAMA Neurol.

[28] Fischer LR (2004). Amyotrophic lateral sclerosis is a distal axonopathy: evidence in mice and man. Exp Neurol.

[29] Dadon-Nachum M, Melamed E, Offen D (2011). The, “Dying-Back” Phenomenon of Motor Neurons in ALS. J Mol Neurosci.

[30] Neumann M (2009). Phosphorylation of S409/410 of TDP-43 is a consistent feature in all sporadic and familial forms of TDP-43 proteinopathies. Acta Neuropathol.

[31] Brettschneider J (2013). Stages of pTDP-43 pathology in amyotrophic lateral sclerosis. Ann Neurol.

[32] Brettschneider J (2014). TDP-43 pathology and neuronal loss in amyotrophic lateral sclerosis spinal cord. Acta Neuropathol.

[33] Braak H, Ludolph A, Thal DR, del Tredici K (2010). Amyotrophic lateral sclerosis: Dash-like accumulation of phosphorylated TDP-43 in somatodendritic and axonal compartments of somatomotor neurons of the lower brainstem and spinal cord. Acta Neuropathol.

[34] Onozato T (2016). Axonal TDP-43 aggregates in sporadic amyotrophic lateral sclerosis. Neuropathol Appl Neurobiol.

[35] Jovičič A (2015). Modifiers of C9orf72 dipeptide repeat toxicity connect nucleocytoplasmic transport defects to FTD/ALS. Nat Neurosci.

[36] Lin YC (2021). Interactions between ALS-linked FUS and nucleoporins are associated with defects in the nucleocytoplasmic transport pathway. Nat Neurosci.

[37] Chou CC, et al. TDP-43 pathology disrupts nuclear pore complexes and nucleocytoplasmic transport in ALS/FTD. Nat Neurosci 2017;21:2 21, 228–239 (2018) 10.1038/s41593-017-0047-3 PMC5800968 29311743

[38] Khosravi B (2017). Cytoplasmic poly-GA aggregates impair nuclear import of TDP-43 in C9orf72 ALS/FTLD. Hum Mol Genet.

[39] Doll SG (2022). Recognition of the TDP-43 nuclear localization signal by importin α1/β. Cell Rep.

[40] Doll SG, Cingolani G (2022). Importin α/β and the tug of war to keep TDP-43 in solution: quo vadis?. Bioessays.

[41] Khalil B (2022). Nuclear import receptors are recruited by FG-nucleoporins to rescue hallmarks of TDP-43 proteinopathy. Mol Neurodegener.

[42] Alami NH (2014). Axonal Transport of TDP-43 mRNA Granules Is Impaired by ALS-Causing Mutations. Neuron.

[43] Fallini C, Bassell GJ, Rossoll W (2012). The ALS disease protein TDP-43 is actively transported in motor neuron axons and regulates axon outgrowth. Hum Mol Genet.

[44] Liao YC (2019). RNA Granules Hitchhike on Lysosomes for Long-Distance Transport, Using Annexin A11 as a Molecular Tether. Cell.

[45] Porta S, et al. Patient-derived frontotemporal lobar degeneration brain extracts induce formation and spreading of TDP-43 pathology in vivo. Nat Commun 2018;9:1 9, 1–15 (2018). 10.1038/s41467-018-06548-9 PMC6181940 30310141

[46] Ding X (2021). Spreading of TDP-43 pathology via pyramidal tract induces ALS-like phenotypes in TDP-43 transgenic mice. Acta Neuropathol Commun.

[47] Smethurst P (2016). In vitro prion-like behaviour of TDP-43 in ALS. Neurobiol Dis.

[48] Nonaka T (2013). Prion-like Properties of Pathological TDP-43 Aggregates from Diseased Brains. Cell Rep.

[49] Iguchi Y (2016). Exosome secretion is a key pathway for clearance of pathological TDP-43. Brain.

[50] Feiler MS (2015). TDP-43 is intercellularly transmitted across axon terminals. J Cell Biol.

[51] Sorarú G (2010). TDP-43 in skeletal muscle of patients affected with amyotrophic lateral sclerosis. Amyotroph Lateral Scler.

[52] Mori F (2019). Phosphorylated TDP-43 aggregates in skeletal and cardiac muscle are a marker of myogenic degeneration in amyotrophic lateral sclerosis and various conditions. Acta Neuropathol Commun.

[53] Ketterer C, Zeiger U, Budak MT, Rubinstein NA, Khurana TS (2010). Identification of the neuromuscular junction transcriptome of extraocular muscle by laser capture microdissection. Invest Ophthalmol Vis Sci.

[54] Riemenschneider H (2022). Gel-like inclusions of C-terminal fragments of TDP-43 sequester stalled proteasomes in neurons. EMBO Rep.

[55] Lu S (2022). Heat-shock chaperone HSPB1 regulates cytoplasmic TDP-43 phase separation and liquid-to-gel transition. Nat Cell Biol.

[56] Ormeño F (2020). Chaperone Mediated Autophagy Degrades TDP-43 Protein and Is Affected by TDP-43 Aggregation. Front Mol Neurosci.

[57] Patel P (2022). Intra-axonal translation of Khsrp mRNA slows axon regeneration by destabilizing localized mRNAs. Nucleic Acids Res.

[58] Rotem N (2017). ALS Along the Axons – Expression of Coding and Noncoding RNA Differs in Axons of ALS models. Sci Rep.

[59] Fazal R (2021). HDAC6 inhibition restores TDP-43 pathology and axonal transport defects in human motor neurons with TARDBP mutations. EMBO J.

[60] Cohen TJ, et al. An acetylation switch controls TDP-43 function and aggregation propensity. Nat Commun 2015;6:1 6, 1–13 (2015). 10.1038/ncomms6845 PMC4407365 25556531

[61] Pirie E (2021). S-nitrosylated TDP-43 triggers aggregation, cell-to-cell spread, and neurotoxicity in hiPSCs and in vivo models of ALS/FTD. Proc Natl Acad Sci U S A.

[62] Eck RJ, Kraemer BC, Liachko NF (2021). Regulation of TDP-43 phosphorylation in aging and disease. Geroscience.

[63] Buratti E (2015). Functional Significance of TDP-43 Mutations in Disease. Adv Genet.

[64] Conicella AE (2020). TDP-43 α-helical structure tunes liquid–liquid phase separation and function. Proc Natl Acad Sci U S A.

[65] Barmada SJ (2010). Cytoplasmic Mislocalization of TDP-43 Is Toxic to Neurons and Enhanced by a Mutation Associated with Familial Amyotrophic Lateral Sclerosis. J Neurosci.

[66] Johnson BS (2009). TDP-43 Is Intrinsically Aggregation-prone, and Amyotrophic Lateral Sclerosis-linked Mutations Accelerate Aggregation and Increase Toxicity. J Biol Chem.

[67] da Silva LAG (2022). Disease-linked TDP-43 hyperphosphorylation suppresses TDP-43 condensation and aggregation. EMBO J.

[68] Nonaka T (2016). Phosphorylation of TAR DNA-binding protein of 43 kDa (TDP-43) by truncated casein kinase 1δ triggers mislocalization and accumulation of TDP-43. J Biol Chem.

[69] Krach F (2018). Transcriptome–pathology correlation identifies interplay between TDP-43 and the expression of its kinase CK1E in sporadic ALS. Acta Neuropathol.

[70] Martínez-González L, et al. Motor neuron preservation and decrease of in vivo TDP-43 phosphorylation by protein CK-1δ kinase inhibitor treatment. Sci Rep 2020;10:1 10, 1–12 (2020). 10.1038/s41598-020-61265-y PMC7064575 32157143

[71] Deng X, et al. CHMP2B regulates TDP-43 phosphorylation and cytotoxicity independent of autophagy via CK1. J Cell Biol 2021;221. 10.1083/jcb.202103033 PMC8570292 34726688

[72] Nozal V (2022). TDP-43 Modulation by Tau-Tubulin Kinase 1 Inhibitors: A New Avenue for Future Amyotrophic Lateral Sclerosis Therapy. J Med Chem.

[73] Tian Y (2021). Tau-tubulin kinase 1 phosphorylates TDP-43 at disease-relevant sites and exacerbates TDP-43 pathology. Neurobiol Dis.

[74] Liachko NF (2013). CDC7 inhibition blocks pathological TDP-43 phosphorylation and neurodegeneration. Ann Neurol.

[75] Davis SA, Gan KA, Dowell JA, Cairns NJ, Gitcho MA (2017). TDP-43 expression influences amyloidβ plaque deposition and tau aggregation. Neurobiol Dis.

[76] Ferri A (2004). Activity of protein phosphatase calcineurin is decreased in sporadic and familial amyotrophic lateral sclerosispatients. J Neurochem.

[77] Liachko NF (2016). The phosphatase calcineurin regulates pathological TDP-43 phosphorylation. Acta Neuropathol.

[78] Gu J (2018). Protein Phosphatase 1 dephosphorylates TDP-43 and suppresses its function in tau exon 10 inclusion. FEBS Lett.

[79] Maraschi AM (2021). SUMOylation Regulates TDP-43 Splicing Activity and Nucleocytoplasmic Distribution. Mol Neurobiol.

[80] Sahoo PK (2018). Axonal G3BP1 stress granule protein limits axonal mRNA translation and nerve regeneration. Nat Commun.

[81] Maday S, Twelvetrees AE, Moughamian AJ, Holzbaur ELF (2014). Axonal Transport: Cargo-Specific Mechanisms of Motility and Regulation. Neuron.

[82] Harrington AW, Ginty DD (2013). Long-distance retrograde neurotrophic factor signalling in neurons. Nat Rev Neurosci.

[83] Saxton WM, Hollenbeck PJ (2012). The axonal transport of mitochondria. J Cell Sci.

[84] Mandal A, Drerup CM (2019). Axonal Transport and Mitochondrial Function in Neurons. Front Cell Neurosci.

[85] Dalla Costa I (2021). The functional organization of axonal mRNA transport and translation. Nat Rev Neurosci.

[86] Villarin JM, McCurdy EP, Martínez JC, Hengst U (2016). Local synthesis of dynein cofactors matches retrograde transport to acutely changing demands. Nat Commun.

[87] Hafner AS, Donlin-Asp PG, Leitch B, Herzog E, Schuman EM. Local protein synthesis is a ubiquitous feature of neuronal pre- And postsynaptic compartments. Science (1979) 364, (2019). 10.1126/science.aau3644 31097639

[88] Kislauskis EH, Zhu X, Singer RH (1997). beta-Actin messenger RNA localization and protein synthesis augment cell motility. J Cell Biol.

[89] Eng H, Lund K, Campenot RB (1999). Synthesis of β-tubulin, actin, and other proteins in axons of sympathetic neurons in compartmented cultures. J Neurosci.

[90] Xing L, Bassell GJ (2013). mRNA localization: an orchestration of assembly, traffic and synthesis. Traffic.

[91] Kiebler MA, Bassell GJ (2006). Neuronal RNA Granules: Movers and Makers. Neuron.

[92] Lukavsky PJ (2013). Molecular basis of UG-rich RNA recognition by the human splicing factor TDP-43. Nat Struct Mol Biol.

[93] Colombrita C (2009). TDP-43 is recruited to stress granules in conditions of oxidative insult. J Neurochem.

[94] Wang I-F, Wu L-S, Chang H-Y, Shen C-KJ (2008). TDP-43, the signature protein of FTLD-U, is a neuronal activity-responsive factor. J Neurochem.

[95] Yu Z (2012). Neurodegeneration-associated TDP-43 interacts with fragile X mental retardation protein (FMRP)/staufen (STAU1) and regulates SIRT1 expression in neuronal cells. J Biol Chemi.

[96] Narayanan RK (2013). Identification of RNA bound to the TDP-43 ribonucleoprotein complex in the adult mouse brain. Amyotroph Lateral Scler Frontotemporal Degener.

[97] Sephton CF (2011). Identification of neuronal RNA targets of TDP-43-containing ribonucleoprotein complexes. J Biol Chem.

[98] Ayala YM (2011). TDP-43 regulates its mRNA levels through a negative feedback loop. EMBO J.

[99] Harbauer AB (2022). Neuronal mitochondria transport Pink1 mRNA via synaptojanin 2 to support local mitophagy. Neuron.

[100] Cohen B (2022). Co-transport of the nuclear-encoded Cox7c mRNA with mitochondria along axons occurs through a coding-region-dependent mechanism. J Cell Sci.

[101] Cioni J-M (2019). Late Endosomes Act as mRNA Translation Platforms and Sustain Mitochondria in Axons Article Late Endosomes Act as mRNA Translation Platforms and Sustain Mitochondria in Axons. Cell.

[102] Wang W (2016). The inhibition of TDP-43 mitochondrial localization blocks its neuronal toxicity. Nat Med.

[103] Magrané J, Cortez C, Gan WB, Manfredi G (2014). Abnormal mitochondrial transport and morphology are common pathological denominators in SOD1 and TDP43 ALS mouse models. Hum Mol Genet.

[104] Wang P (2019). TDP-43 induces mitochondrial damage and activates the mitochondrial unfolded protein response. PLoS Genet.

[105] Terenzio M (2018). Locally translated mTOR controls axonal local translation in nerve injury. Science.

[106] Cosker KE, Pazyra-Murphy MF, Fenstermacher SJ, Segal RA. Target-derived neurotrophins coordinate transcription and transport of Bclw to prevent axonal degeneration. Ann Intern Med 2013;158. 10.1523/JNEUROSCI.3862-12.2013 PMC3866501 23516285

[107] Lautenschläger J (2022). Protein phase separation hotspots at the presynapse. Open Biology.

[108] Chen X, Wu X, Wu H, Zhang M (2020). Phase separation at the synapse. Nat Neurosci.

[109] Tsang B (2019). Phosphoregulated FMRP phase separation models activity-dependent translation through bidirectional control of mRNA granule formation. Proc Natl Acad Sci U S A.

[110] So E (2018). Mitochondrial abnormalities and disruption of the neuromuscular junction precede the clinical phenotype and motor neuron loss in hFUSWT transgenic mice. Hum Mol Genet.

[111] Gopal PP, Nirschl JJ, Klinman E, Holzbaurb ELF (2017). Amyotrophic lateral sclerosis-linked mutations increase the viscosity of liquid-like TDP-43 RNP granules in neurons. Proc Natl Acad Sci U S A.

[112] Liu-Yesucevitz L (2010). Tar DNA binding protein-43 (TDP-43) associates with stress granules: Analysis of cultured cells and pathological brain tissue. PLoS One.

[113] Aulas A, Stabile S, Vande Velde C (2012). Endogenous TDP-43, but not FUS, contributes to stress granule assembly via G3BP. Mol Neurodegener.

[114] Russo A (2017). Increased cytoplasmic TDP-43 reduces global protein synthesis by interacting with Rack1 on polyribosomes. Hum Mol Genet.

[115] Neelagandan N (2019). TDP-43 enhances translation of specific mRNAs linked to neurodegenerative disease. Nucleic Acids Res.

[116] Coyne AN (2015). Fragile X protein mitigates TDP-43 toxicity by remodeling RNA granules and restoring translation. Hum Mol Genet.

[117] Gioio AE (2001). Local synthesis of nuclear-encoded mitochondrial proteins in the presynaptic nerve terminal. J Neurosci Res.

[118] Hillefors M, Gioio AE, Mameza MG, Kaplan BB (2007). Axon viability and mitochondrial function are dependent on local protein synthesis in sympathetic neurons. Cell Mol Neurobiol.

[119] Maciel R (2018). The human motor neuron axonal transcriptome is enriched for transcripts related to mitochondrial function and microtubule-based axonal transport. Exp Neurol.

[120] Wang W (2013). The ALS disease-associated mutant TDP-43 impairs mitochondrial dynamics and function in motor neurons. Hum Mol Genet.

[121] Shigeoka T (2019). On-Site Ribosome Remodeling by Locally Synthesized Ribosomal Proteins in Axons. Cell Rep.

[122] von Kügelgen N, Chekulaeva M (2020). Conservation of a core neurite transcriptome across neuronal types and species. Wiley Interdiscip Rev: RNA.

[123] Nagano S (2020). TDP-43 transports ribosomal protein mRNA to regulate axonal local translation in neuronal axons. Acta Neuropathol.

[124] Bartel DP (2018). Metazoan MicroRNAs. Cell.

[125] Gershoni-Emek N (2018). Localization of RNAi Machinery to Axonal Branch Points and Growth Cones Is Facilitated by Mitochondria and Is Disrupted in ALS. Front Mol Neurosci.

[126] Sasaki Y, Gross C, Xing L, Goshima Y, Bassell GJ. Identification of axon-enriched MicroRNAs localized to growth cones of cortical neurons. Dev Neurobiol. 2013. 10.1002/dneu.22113 10.1002/dneu.22113 PMC4111797 23897634

[127] Andreassi C (2021). Cytoplasmic cleavage of IMPA1 3′ UTR is necessary for maintaining axon integrity. Cell Rep.

[128] Wang B, Bao L (2017). Axonal microRNAs: Localization, function and regulatory mechanism during axon development. J Mol Cell Biol.

[129] Corradi E, Baudet ML (2020). In the right place at the right time: Mirnas as key regulators in developing axons. Int J Mol Sci.

[130] Corradi E (2020). Axonal precursor mi RNA s hitchhike on endosomes and locally regulate the development of neural circuits. EMBO J.

[131] Kawahara Y, Mieda-Sato A (2012). TDP-43 promotes microRNA biogenesis as a component of the Drosha and Dicer complexes. Proc Natl Acad Sci U S A.

[132] Buratti E (2010). Nuclear factor TDP-43 can affect selected microRNA levels. FEBS J.

[133] Paez-Colasante X (2020). Cytoplasmic TDP43 Binds microRNAs: New Disease Targets in Amyotrophic Lateral Sclerosis. Front Cell Neurosci.

[134] Leung AKL, Calabrese JM, Sharp PA (2006). Quantitative analysis of argonaute protein reveals microRNA-dependent localization to stress granules. Proc Natl Acad Sci U S A.

[135] Pare JM (2009). Hsp90 regulates the function of argonaute 2 and its recruitment to stress granules and P-bodies. Mol Biol Cell.

[136] Zuo X (2021). TDP-43 aggregation induced by oxidative stress causes global mitochondrial imbalance in ALS. Nat Struct Mol Biol.

[137] Sleigh JN (2020). Mice Carrying ALS Mutant TDP-43, but Not Mutant FUS, Display In Vivo Defects in Axonal Transport of Signaling Endosomes. Cell Rep.

[138] Chen Y, Cohen TJ (2019). Aggregation of the nucleic acid– binding protein TDP-43 occurs via distinct routes that are coordinated with stress granule formation. J Biol Chem.

[139] Ling JP, Pletnikova O, Troncoso JC, Wong PC (2015). TDP-43 repression of nonconserved cryptic exons is compromised in ALS-FTD. Science.

[140] Ma XR (2022). TDP-43 represses cryptic exon inclusion in the FTD–ALS gene UNC13A. Nature.

[141] Cook C, Petrucelli L (2019). Genetic Convergence Brings Clarity to the Enigmatic Red Line in ALS. Neuron.

[142] Mathieu C, Pappu RV, Taylor JP. Beyond aggregation: Pathological phase transitions in neurodegenerative disease. Science (1979) 370, (2020). 10.1126/science.abb8032 PMC8359821 33004511

[143] Portz B, Lee BL, Shorter J (2021). FUS and TDP-43 Phases in Health and Disease. Trends Biochem Sci.

[144] Molliex A (2015). Phase Separation by Low Complexity Domains Promotes Stress Granule Assembly and Drives Pathological Fibrillization. Cell.

[145] Mann JR (2019). RNA Binding Antagonizes Neurotoxic Phase Transitions of TDP-43. Neuron.

[146] Conicella AE, Zerze GH, Mittal J, Fawzi NL (2016). ALS Mutations Disrupt Phase Separation Mediated by α-Helical Structure in the TDP-43 Low-Complexity C-Terminal Domain. Structure.

[147] McGurk L (2018). Poly(ADP-Ribose) Prevents Pathological Phase Separation of TDP-43 by Promoting Liquid Demixing and Stress Granule Localization. Mol Cell.

[148] Zhang X (2020). In vivo stress granule misprocessing evidenced in a FUS knock-in ALS mouse model. Brain.

[149] Dubinski A, et al. Stress granule assembly in vivo is deficient in the CNS of mutant TDP-43 ALS mice. Hum Mol Genet.2022 10.1093/HMG/DDAC206 10.1093/hmg/ddac206 PMC9840205 35994036

[150] Buratti E (2021). Targeting TDP-43 proteinopathy with drugs and drug-like small molecules. British Journal of Pharmacology.

